# Risk factors for systemic lupus erythematosus complicated with tuberculosis infection: meta-analyses and systematic reviews

**DOI:** 10.7717/peerj.20448

**Published:** 2026-01-13

**Authors:** Xiaoyi Zhang, Hai Zheng, Peng Zhou, Wenfeng Hu, Yuxin Si, Xianhui Wu, Chen Shen

**Affiliations:** 1 The First Clinical Medical School, Hubei University of Chinese Medicine, Wuhan, Wuhan, China; 2 Department of Neurology, Wan Mizhai Hospital of Luotian County, Huanggang, China; 3 School of Clinical Traditional Chinese Medicine, Hubei University of Chinese Medicine, Wuhan, China; 4 Hubei Provincial Hospital of Traditional Chinese Medicine, Hubei Shizhen Laboratory, Affiliated Hospital of Hubei University of Chinese Medicine, Wuhan, China

**Keywords:** Systemic lupus erythematosus, TB infection, Risk factor, Meta-analysis, Systematic review

## Abstract

**Background:**

To unravel the risk factors of systemic lupus erythematosus (SLE) complicated with tuberculosis (TB) infection through a systematic review and meta-analysis.

**Methods:**

PubMed, Embase, Cochrane Library, and Web of Science databases were searched for relevant research articles on systemic lupus erythematosus with TB infection from inception to June 12, 2024. Analyses of the data were performed with Stata 15.0.

**Results:**

The analysis incorporated 19 articles, comprising nine case-control and 10 cohort studies. In these studies, 1,292 patients with SLE complicated with TB infection and 5,703 SLE patients without TB infection were evaluated. The meta-analysis findings elucidated several pivotal risk factors with statistical significance: male (odds ratio (OR) = 1.32, 95% confidence interval (CI) [1.06–1.64], probability value (*P*) = 0.011), lymphocytopenia (OR = 2.65, 95% CI [1.98–3.55], *P* = 0.000), anemia (OR = 2.53, 95% CI [1.11–5.77], *P* = 0.001), hypoalbuminemia (OR = 3.46, 95% CI [1.26–9.50], *P* = 0.016), diabetes (OR = 3.05, 95% CI [1.63–5.71], *P* = 0.000). Results of multivariate analysis identified lymphocytopenia (OR = 2.90, 95% CI [1.89–4.45], *P* = 0.000), cumulative glucocorticoids dosage (OR = 4.88, 95% CI [1.85–12.91], *P* = 0.001), and a history of TB exposure (OR = 3.38, 95% CI [1.16–9.86], *P* = 0.026) as risk factors for SLE complicated with TB infection.

**Conclusion:**

Based on available evidence, males, lymphocytopenia, anemia, hypoalbuminemia, diabetes, cumulative glucocorticoids dosage, and the TB exposure history are risk factors for SLE complicated with TB infection.

**PROSPERO registry number:**

CRD42024583278.

## Introduction

Systemic lupus erythematosus (SLE) is a chronic autoimmune disease characterized by immune system dysregulation and multi-organ damage. Its pathogenesis is complex, involving interactions between genetic susceptibility, environmental triggers, hormonal imbalance, and metabolic abnormalities (Kamen, 2014).

Currently, glucocorticoids (GCs) and immunosuppressants remain the cornerstone of SLE treatment. Although these regimens have significantly improved patients’ long-term prognosis, they have also led to a sharp increase in the risk of immunosuppression-related complications. Among these, tuberculosis (TB) infection has become an important trigger for morbidity and mortality in SLE patients (Fayyaz et al., 2015; Kang & Park, 2003; Muñoz-Grajales et al., 2023; Tam et al., 2002). Existing clinical observations indicate that SLE patients have a significantly higher risk of TB infection compared to the general population. This phenomenon is closely related to the inherent immune dysregulation of the disease and the immunosuppressive state associated with treatment. Specifically, SLE patients experience decreased anti-TB immune response capability due to T lymphocyte dysfunction, impaired phagocytic cell activity, and cytokine network imbalance. The long-term use of high-dose glucocorticoids and immunosuppressants further inhibits macrophage-mediated pathogen clearance and granuloma formation, creating a “immunosuppression-infection” vicious cycle. Additionally, metabolic abnormalities (such as hypoalbuminemia, diabetes) may weaken the host’s nutritional barrier and inflammatory regulation ability, causing immune disorders that make SLE patients prone to opportunistic infections and amplify the invasive potential of Mycobacterium TB (Alarcón, 2006; Kang & Park, 2003; Pego-Reigosa et al., 2021). TB infection infection/disease remains one of the most common and life-threatening infectious diseases in SLE patients (Dahlia Abd El-Mohsen et al., 2017; Mathian, Arnaud & Ruiz-Irastorza, 2024). It can induce severe systemic symptoms in patients, and even fatalities and widespread epidemics of the disease (Balbi et al., 2018; Darmawan et al., 2024). Considering the early onset and high prevalence of SLE, identifying risk factors for TB infection is one of the important issues requiring timely and appropriate treatment, which seriously impacts disease outcomes. Although several studies have attempted to identify these risk factors, many results have been found to be inconsistent due to the heterogeneity and complexity of SLE. From this perspective, this study provides a systematic review and meta-analysis to summarize and elucidate the risk factors for TB infection in SLE patients.

## Materials and Methods

The analysis method was developed in accordance with the Preferred Reporting Items for Systematic Review and Meta-analysis Protocols (PRISMA-P) guidelines. The PRISMA guidelines were adhered to throughout the review process. PROSPERO registry number: CRD42024583278.

PubMed, Embase, Cochrane Library, and Web of Science databases were searched for relevant research articles on SLE with TB infection from inception to June 12, 2024. The search was conducted using subject headings plus free terms: systemic lupus erythematosus, tuberculosis OR TB infection, risk factors. Specific search strategies are detailed in Supplemental Material 1.

### Inclusion and exclusion criteria

Inclusion criteria: (1) Study population: patients diagnosed with systemic lupus erythematosus; (2) Exposure factors: TB infection. (3) Study types: case-control studies, cohort studies, or cross-sectional studies; (4) Primary outcome measure: univariate and multivariate risk factor analysis. There was no limitation on univariate and multivariate analyses, but when both univariate and multivariate results were included in the same document, the multivariate results were prioritized, and the multivariate adjusted risk values were extracted. If only univariate analysis was available, the univariate results were extracted.

Criteria for exclusion: Conference abstracts, meta-analyses, protocols, letters, duplicates, systematic reviews, studies without full text, studies with unusable data, and animal experiments.

### Extraction of data

The literature was independently screened by two reviewers (Xiaoyi Zhang and Peng Zhou) to extract data. This entailed reading the titles, abstracts, and complete texts. For literature that could be easily judged, direct screening was performed. For literature with disagreements on inclusion, opinions were sought from the third reviewer (Chen Shen), and the complete manuscript was downloaded and read for screening, which was performed by strictly adhering to the inclusion and exclusion criteria. Related measures in the study were gathered. Cross-checking of the extracted data was implemented to warrant consistency. The main extracted information encompassed the first author’s name, publication year, study design, sample size, country, gender, and age.

### Quality assessment

The quality assessment was conducted as previously described in the study by Zhou et al. (2025). The Newcastle-Ottawa Scale (NOS) was employed to appraise the quality of case-control studies (Stang, 2010), encompassing selection of the study population (four points), comparability of the groups (two points), and exposure factors or outcome measures (three points). The maximum score was 9. A score of ≤4 indicated low quality, 5–6 indicated moderate quality, and ≥7 indicated high quality. Dissents, if any, were discussed or resolved by a third party.

### Statistical analysis

Stata 15.0 was used for statistical analysis. The continuous variables were expressed as weighted mean difference (WMD) and 95% confidence interval (CI), and the dichotomous variables were expressed as odds ratio (OR). For the multivariate analysis, this study pooled the OR and their 95% CI from each included study. Probability value (*P*) value and I-squared (I^2^) were used to judge the heterogeneity among the studies. If necessary, third-party reviewers will recheck the consistency of the data with regard to the handling of missing data. If the data is missing or incomplete, we will contact the research author to obtain the data. Last but not least, studies without available data will be excluded.

## Results

### Literature search process and results

**Figure 1:**
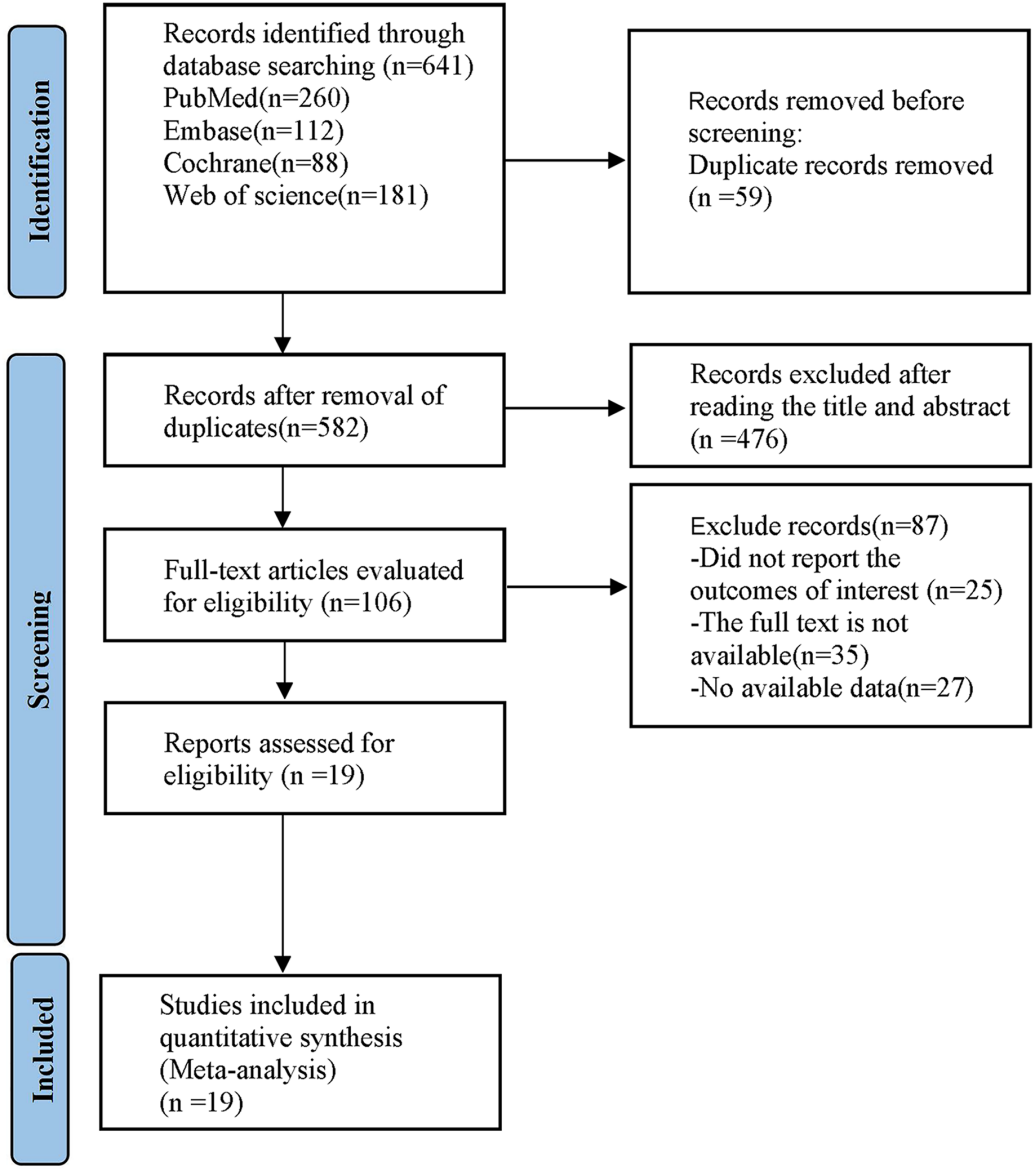
PRISMA flow diagram of the study process.

After conducting a preliminary search of the databases of PubMed, Embase, Cochrane Library, and Web of Science, a total of 641 articles were found. Following the elimination of duplicates, there were 582 articles left. Through the screening of titles and abstracts, this number was decreased to 476 articles, and following the assessment of the entire texts, 19 studies were included. The specific search process is illustrated in Fig. 1.

### Basic characteristics of the included studies

Of the 19 included articles, nine were case-control studies (Damara, Ariane & Winston, 2022; González-Naranjo et al., 2021; Hodkinson, Musenge & Tikly, 2009; Kumar et al., 2022; Tam et al., 2002; Torres-González et al., 2018; Yang et al., 2007; Yun et al., 2002; Zhang, Wang & Ma, 2008) and 10 were cohort studies (Chen et al., 2012; Ghosh, Patwardhan & Pradhan, 2009; Hamijoyo et al., 2022; Lao et al., 2019; Mok et al., 2007; Muhammed et al., 2021; Pasoto et al., 2010; Sayarlioglu et al., 2004; Zhang et al., 2023a, 2023b), including 527 individuals with SLE complicated with TB infection and 2,167 SLE patients without TB infection. The ages of participants in the included studies ranged from 9 to 42 years. Specific characteristics of the literature are presented in Table 1. The 19 articles included were evaluated using the NOS scale, with 10 articles scoring seven points, and nine articles scoring eight points. The overall quality of the studies included was high. Detailed quality assessment results are presented in Table 2.

**Table 1:** Characteristics of included studies.

Study	Country	Study design	Sample size	Number of TB cases	Sex (M/F)	tb	ntb	Diagnostic criteria	Regression model
Lao et al. (2019)	Southern China	Cohort study	177	59	36/141	35		TB: (1) typical radiological manifestations, (2) positive *M. tuberculosis* culture, or (3) the presence of caseous necrosis in biopsied specimen. SLE: 2012 criteria of SLE (ACR)	Logistic regression
Damara, Ariane & Winston (2022)	Indonesia	Case-control study	48	24	2/46	12–41	16–46	TB: The diagnosis of tuberculosis infection in this study was made by using acid-fast bacilli test, Mantoux test, GeneXpert test, magnetic resonance imaging (MRI), CT scan, spinal tap, biopsy, X-ray with clinical diagnosis, and polymerase chain reaction (PCR). SLE: meeting the 1997 American College of Rheumatology (ACR) SLE classification criteria	Logistic regression
Chen et al. (2012)	China	Cohort study	36	19	4/32	41		TB: SLE patients with active tuberculosis infection (n019) were identified if Tuberculosis or acid-fast staining bacilli was recovered from specimens, or supported by clinical and/or radiological features and/or positive enzyme linked immunospot assay for *M. tuberculosis* and/or response to anti-Tuberculosis therapy SLE: meeting the 1997 American College of Rheumatology (ACR) SLE classification criteria	Logistic regression
Ghosh, Patwardhan & Pradhan (2009)	India	Cohort study	70	14	10/60	9–36		TB: Tuberculosis as evidenced by clinical features, sputum positivity for acid fast bacilli and positive chest X-ray were also included in this study. We have used strict criteria where either acid fast bacilli had been demonstrated in the sputum or a biopsy specimen had shown classical caseating granuloma for the diagnosis of the tuberculosis. SLE: 1997 American College of Rheumatology (ACR) SLE classification criteria	
González-Naranjo et al. (2021)	Colombian	Case-control study	268	67	39/229	29		TB: Chest radiographic findings, positive *Mycobacterium tuberculosis* culture, a positive sputum smear microscopy, a positive polymerase chain reaction (PCR) assay for *M. tuberculosis* complex, or histopathological findings compatible with TB SLE: 1997 American College of Rheumatology (ACR) SLE classification criteria	Logistic regression
Sayarlioglu et al. (2004)	Turkish	Cohort study	116	20	10/106	15–52	11–60	TB: The diagnosis of TB was confirmed with clinical, radiological and laboratory investigations including Ziehl–Nielsen staining or culture on Lowenstein–Jensen media of sputum or joint/soft tissue aspirate. Diagnosis of TB was confirmed microbiologically in 65% of patients. SLE: 未提及	
Tam et al. (2002)	Hong Kong	Case-control study	57	19	6/51	29–42.3	28.5–44.3	TB: The diagnosis of TB was confirmed by one of the followings: a positive acid-fast bacillus(AFB) smear, a positive culture of Mycobacterium TB from appropriate clinical specimens, or a characteristic histopathological examination of caseating granuloma on appropriate surgical specimens. SLE: Updating the American College of Rheumatology revised criteria for the classification of systemic lupus erythematosus. Arthritis Rheum 1997;40:1725.	Logistic regression
Zhang, Wang & Ma (2008)	China	Case-control study	452	42		35		TB: Diagnostic criteria for tuberculosis: clinical manifestations of infection and at least one of the following conditions: (1) positive results of sputum or bronchoscopic brushing, pleural effusion, cerebrospinal fluid, joint fluid smear or culture of *Mycobacterium tuberculosis*; (2) pathological biopsy of the affected lesion shows caseous necrosis and/or tuberculous granuloma; (3) typical imaging manifestations and effective anti-tuberculosis treatment; (4) in the absence of a clear tuberculosis infection focus, when increased doses of hormones and routine use of strong antibiotics that cover both Gram-negative and Gram-positive bacteria are ineffective, clinical manifestations are relieved and laboratory auxiliary tests show normal results after anti-tuberculosis treatment. SLE: Updating the American College of Rheumatology revised criteria for the classification of systemic lupus erythematosus. Arthritis Rheum 1997;40:1725.	
Torres-González et al. (2018)	Mexico	Case-control study	144	72	16/128	24–44	24–45.5	TB: We established the TB diagnosis with the presence of clinical symptoms in addition to one or more of the following: a positive culture for *Mycobacterium tuberculosis* complex, a positive direct smear microscopy, a positive PCR for TB (TIBMOLBIOL, Berlin Germany, or GeneXpert, CepheidSunnyvale CA), histopathological findings compatible with TB, chest radiography findings, diagnostic levels of adenosine deaminase in fluid samples, or clinical response to anti-TB drugs. Two infectious diseases specialists (PTG and APdL) reviewed the medical records to confirm the TB diagnosis. SLE: Systemic lupus erythematosus disease activity index 2000.	Logistic regression
Hamijoyo et al. (2022)	Indonesia	Cohort study	1,278	131	55/1,223	25		TB: Histopathology, sputum smear microscopy for acid-fast bacilli, and sputum molecular TB diagnostic testing by Xpert MTB/Rif (which was introduced in approximately 2012) were used to define a diagnosis of definite TB, Clinical TB (without microbiological confirmation) was de-fined as TB diagnosed or anti-TB treatment started by the at tending physician. SLE: patients were included if they fulfilled American College of Rheumatology 1997 and/or Systemic Lupus International Collaborating Clinics 2012 Classification Criteria for SLE	Cox regression
Hodkinson, Musenge & Tikly (2009)	South Africa	Case-control study	291	97	30/261	35		TB: The diagnosis of TB was based on direct microscopy of sputum and fluid using either the Ziehl Nielsen or auramine staining methods or mycobacterial culture, histology or typical chest X-ray (CXR) findings in the setting of suggestive constitutional symptoms. SLE: 1997 American College of Rheumatology (ACR) SLE classification criteria	Logistic regression
Mok et al. (2007)	Hong Kong	Cohort study	50	39	6/44	22–78	23–69	TB: Cases of TM infections are ascertained with a positive culture and documented follow-up were identified. SLE: The 1982 revised criteria forthe classification of systemic lupus erythematosus.	Logistic regression
Zhang et al. (2023a)	China	Cohort study	1,361	16	101/1,260	25–49	28–45	SLE: 1997 American College of Rheumatology (ACR) SLE classification criteria	Cox regression
Muhammed et al. (2021)	Indian	Cohort study	1,335	48	93/1,242	32		TB: A diagnosis of active tuberculosis required bacteriological [culture positive, demonstration of acid-fast bacilli or polymerase chain reaction (PCR) positivity] or histopathological evidence suggestive of tuberculosis or CT/MRI suggestive of tuberculosis and initiation of four drug anti tuberculous therapy SLE: All patients fulflled 1997 ACR and/or SLICC 2012 classifcation criteria for SLE.	
Pasoto et al. (2010)	Sao Paulo-SP-Brazil	Cohort study	60	20	0/60	42		TB: Pulmonary TB diagnosis was confirmed by the presence of clinical manifestations (fever, cough, sputum, dyspnea, hemoptysis, and weight loss) plus one of these findings: a positive acid-fast bacillus and/or a positive culture of *Mycobacterium tuberculosis* from smear or tissue specimens, or a characteristic histopathological pattern of caseating granuloma on tissue specimens (TBþ group). SLE: Updating the American College of Rheumatology revised criteria for the classification of systemic lupus erythematosus. Arthritis Rheum 1997	
Kumar et al. (2022)	India	Case-control study	123	29	8/115	21–36	21–35	TB: TB was defined as either microbiologically proven or clinically diagnosed. Microbiologically confirmed TB case refers to patients with any of their biological specimen positive for Acid-fast bacilli (AFB) staining or positive on culture or positive through a quality-assured rapid diagnostic molecular test such as polymerase chain reaction or cartridge-based nucleic acid amplification test (CBNAAT). Clinically diagnosed TB case refers to a presumptive TB who does not satisfy the above microbiological criteria but has been diagnosed by the treating clinician based on imaging abnormalities, histopathology, or clinical signs with an intention to treat the patient with the entire course of anti-TB treatment (ATT). SLE: SLE disease flare was defined according to SELENA-SLEDAI	
Yang et al. (2007)	China	Case-control study	25	19	1/37	31		SLE: (1982) The revised criteria for the classification of systemic lupus erythematosus. Arthritis Rheum	
Zhang et al. (2023b)	China	Cohort study	2,918	2,229	217/2,701	38		TB: The IGRA is recommended for use as the preferred method for screening for LTBI in patients with rheumatic diseases, due to its higher accuracy compared to the TST. SLE: Systemic Lupus Erythematosus Disease Activity Index 2000 (SLEDAI-2K)	Logistic regression
Yun et al. (2002)	Korea	Case-control study	283	15	15/268	37		TB: tuberculosis infection was defined as the newly developed symptoms and/or signs associated with one of the following results: (1) identification of acid fast organisms by specimen smear, culture and/or polymerase chain reaction (PCR); (2) typical histological findings at the involved site; or (3) typical chest x-ray findings which improved after anti-tuberculosis treatment. SLE: The patients enrolled fulfilled the 1982 American College of Rheumatology (ACR) revised criteria for SLE (10) and the 1987 ACR revised criteria for RA	

**Table 2:** NOS rating scale.

Case control									
**Study**	Is the case definition adequate?	Representativeness of the cases	Determination of control group	Definition of controls	Comparability of cases and controls based on the design or analysis	Ascertainment of exposure	Same method of ascertainment for cases and controls	Non response	**Total scores**
Damara, Ariane & Winston (2022)	*	*	*	*		*	*	*	7
González-Naranjo et al. (2021)	*	*	*	*	*	*	*	*	8
Tam et al. (2002)	*	*	*	*	*	*	*	*	8
Torres-González et al. (2018)	*	*	*		*	*	*	*	7
Zhang, Wang & Ma (2008)	*	*	*	*	*	*	*	*	8
Hodkinson, Musenge & Tikly (2009)	*	*	*	*	*	*	*	*	8
Kumar et al. (2022)	*	*	*	*	*	*	*	*	8
Yang et al. (2007)	*	*	*	*	*	*	*	*	8
Yun et al. (2002)	*	*	*	*		*	*	*	7

Cohort study									
Study	Representativeness of the exposed group	Selection of non-exposed groups	Determination of exposure factors	Identification of outcome indicators not yet to be observed at study entry	Comparability of exposed and unexposed groups considered in design and statistical analysis	design and statistical analysis	Adequacy of the study’s evaluation of the outcome	Adequacy of follow-up in exposed and unexposed groups	Total scores
Lao et al. (2019)	*	*	*	*	*	*	*	*	8
Chen et al. (2012)	*	*	*	*		*	*	*	7
Ghosh, Patwardhan & Pradhan (2009)	*	*	*	*		*	*	*	7
Sayarlioglu et al. (2004)	*	*	*	*		*	*	*	7
Hamijoyo et al. (2022)	*	*	*	*	*	*	*	*	8
Mok et al. (2007)	*	*	*	*		*	*	*	7
Zhang et al. (2023b)	*	*	*	*	*	*	*	*	8
Muhammed et al. (2021)	*	*	*	*		*	*	*	7
Pasoto et al. (2010)	*	*	*	*		*	*	*	7
Zhang et al. (2023b)	*	*	*	*		*	*	*	7

### Univariate meta-analysis

#### Male

**Figure 2:**
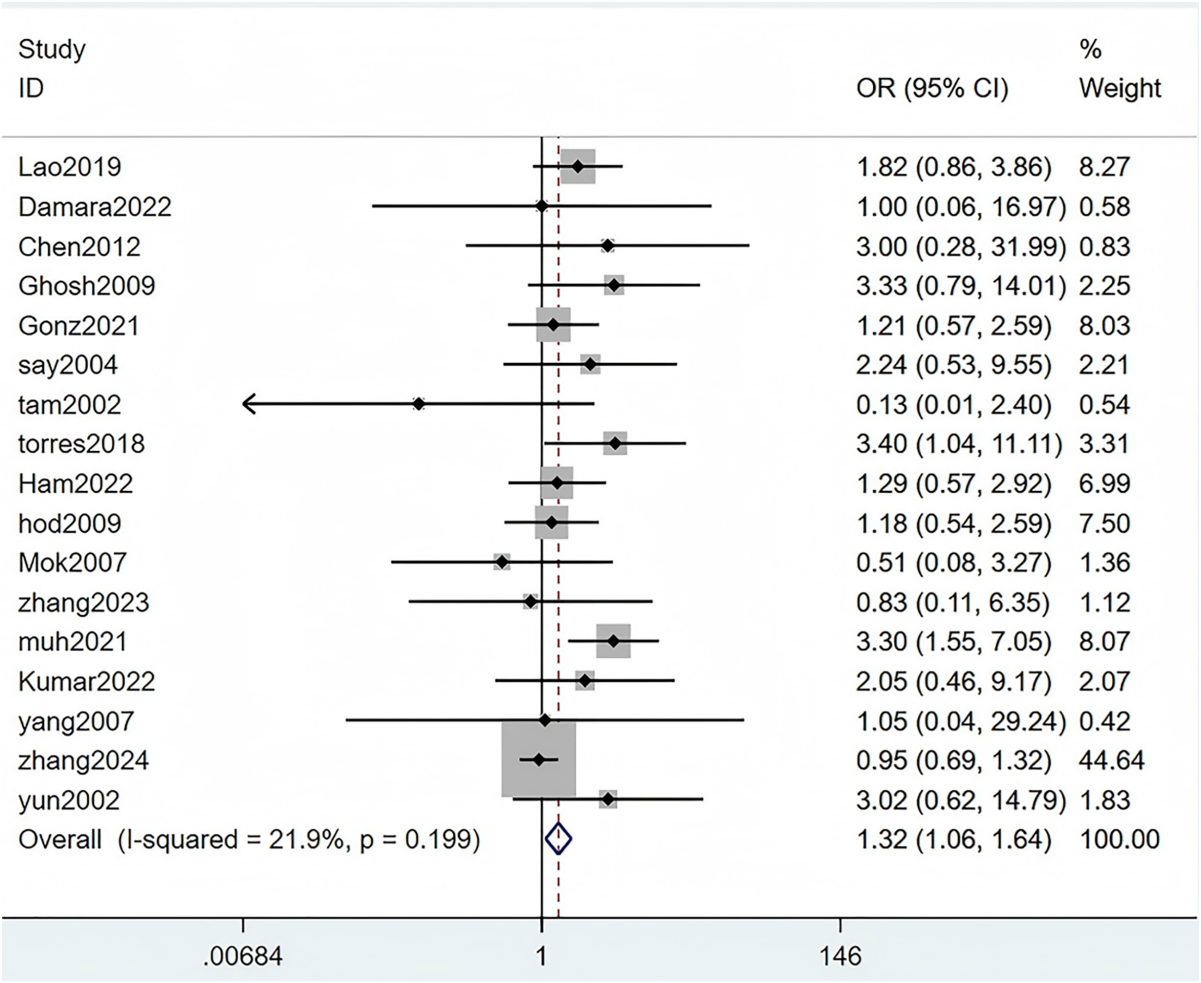
Forest plot of male (Lao et al., 2019; Damara, Ariane & Winston, 2022; Chen et al., 2012; Ghosh, Patwardhan & Pradhan, 2009; González-Naranjo et al., 2021; Sayarlioglu et al., 2004; Tam et al., 2002; Torres-González et al., 2018; Hamijoyo et al., 2022; Hodkinson, Musenge & Tikly, 2009; Mok et al., 2007; Zhang et al., 2023a, 2023b; Muhammed et al., 2021; Kumar et al., 2022; Yang et al., 2007; Zhang, Wang & Ma, 2008; Yun et al., 2002).

Seventeen studies mentioned a history of male gender. The heterogeneity test (I^2^ = 21.9%, *P* = 0.199) was performed through a fixed-effects model. The results uncovered that male was a risk factor for SLE complicated with TB infection, with statistically significant differences (OR = 1.32, 95% CI [1.06–1.64], *P* = 0.011). Details are specified in Fig. 2 and Table 3.

**Table 3:** Single factor analysis table.

Risk factors	No of study	Heterogeneity		OR/WMD (95% CI)	*P*	Egger
		I^2^ (%)	*P*			
male	17	21.9	0.199	1.32 [1.06–1.64]	0.011	0.257
Age at onset of SLE	15	62.6	0.001	−0.38 [−1.90 to 1.13]	0.620	0.935
Duration of SLE	11	76.5	0.000	0.45 [−5.11 to 6.01]	0.873	0.390
Age at diagnosis of tuberculosis	5	0.0	0.497	−0.94 [−2.19 to 0.30]	0.137	0.633
Activity index DAI	9	78.0	0.000	−0.48 [−1.38 to 0.41]	0.290	0.812
Leukopenia	6	63.7	0.017	1.10 [0.56–2.14]	0.786	0.965
Lymphopenia	7	40.9	0.118	2.65 [1.98–3.55]	0.000	0.190
Anemia	5	68.3	0.013	2.53 [1.11–5.77]	0.028	0.830
Hypoalbuminemia	5	84.6	0.000	3.46 [1.26–9.50]	0.016	0.177
Positive anti-nuclear antibody	5	0.0	0.509	1.01 [0.57–1.79]	0.961	0.525
Anti-dsDNA	6	68.9	0.007	0.84 [0.46–1.51]	0.558	0.969
Reduced complement C3	7	63.7	0.011	1.16 [0.74–1.81]	0.528	0.885
Lymphocyte count	4	99.4	0.000	−163.86 [−510.72 to 182.99]	0.354	0.496
CRP	4	97.6	0.000	15.23 [−15.83 to 46.28]	0.337	0.654
Accumulative glucocorticoid	5	89.7	0.000	0.62 [−0.35 to 1.59]	0.213	0.929
Cyclophosphamide	8	97.0	0.000	0.59 [0.14–2.43]	0.464	0.458
Mycophenolate mofetil	7	35.3	0.159	1.21 [1.00–1.47]	0.054	0.316
Methotrexate	7	90.2	0.000	0.44 [0.14–1.37]	0.157	0.675
Cyclosporine A	5	94.5	0.000	0.81 [0.07–8.87]	0.861	0.138
Antiperiodic	3	90.0	0.000	0.34 [0.08–1.43]	0.142	0.512
Hemopathy	6	67.8	0.008	0.78 [0.44–1.40]	0.410	0.547
Diabetes	3	16.6	0.302	3.05 [1.63–5.71]	0.000	0.924
Nephritis	10	98.0	0.000	0.97 [0.21–4.47]	0.966	0.013
Discoid rash	4	0.0	0.901	0.70 [0.34–1.46]	0.342	0.827
Light sensitivity	4	0.0	0.537	0.74 [0.48–1.13]	0.161	0.025
Malar rash	4	0.0	0.567	0.86 [0.58–1.30]	0.484	0.930
Nervous system manifestation	9	33.4	0.151	1.30 [0.90–1.88]	0.165	0.892
Dental ulcer	5	54.4	0.067	1.00 [0.52–1.91]	0.993	0.620
Musculoskeletal involvement	4	0.0	0.701	1.02 [0.66–1.59]	0.917	0.014
Mucocutaneous involvement	5	18.7	0.295	0.75 [0.54–1.05]	0.094	0.811
Blood platelet	3	0.0	1.000	0.00 [−1.26 to 1.26]	1.000	
Corticosteroid dose	4	98.6	0.000	0.55 [0.03–11.25]	0.701	0.118
Vasculitis	3	85.4	0.001	0.23 [0.01–4.05]	0.313	0.297
Arthritis	5	0.0	0.577	1.16 [0.84–1.59]	0.369	0.013
Orrhomeningitis	4	31.9	0.221	1.41 [0.87–2.27]	0.160	0.507
Pleuritis	4	75.3	0.007	2.16 [0.80–5.86]	0.129	0.029
Lung involvement	3	20.6	0.284	1.12 [0.60–2.08]	0.731	0.547
Imidazole hydroxyprine	8	97.6	0.000	0.44 [0.10–1.93]	0.280	0.288
Tuberculosis-related deaths	4	20.0	0.290	0.97 [0.45–2.10]	0.946	0.054
Methylprednisolone pulse within 6 months	4	84.6	0.000	0.25 [0.02–3.87]	0.321	0.201
History of exposure to tuberculosis	7	96.4	0.000	2.23 [0.37–13.50]	0.383	0.260
Other immunosuppressants	5	0.0	0.420	0.91 [0.68–1.22]	0.524	0.769

#### Lymphocytopenia

**Figure 3:**
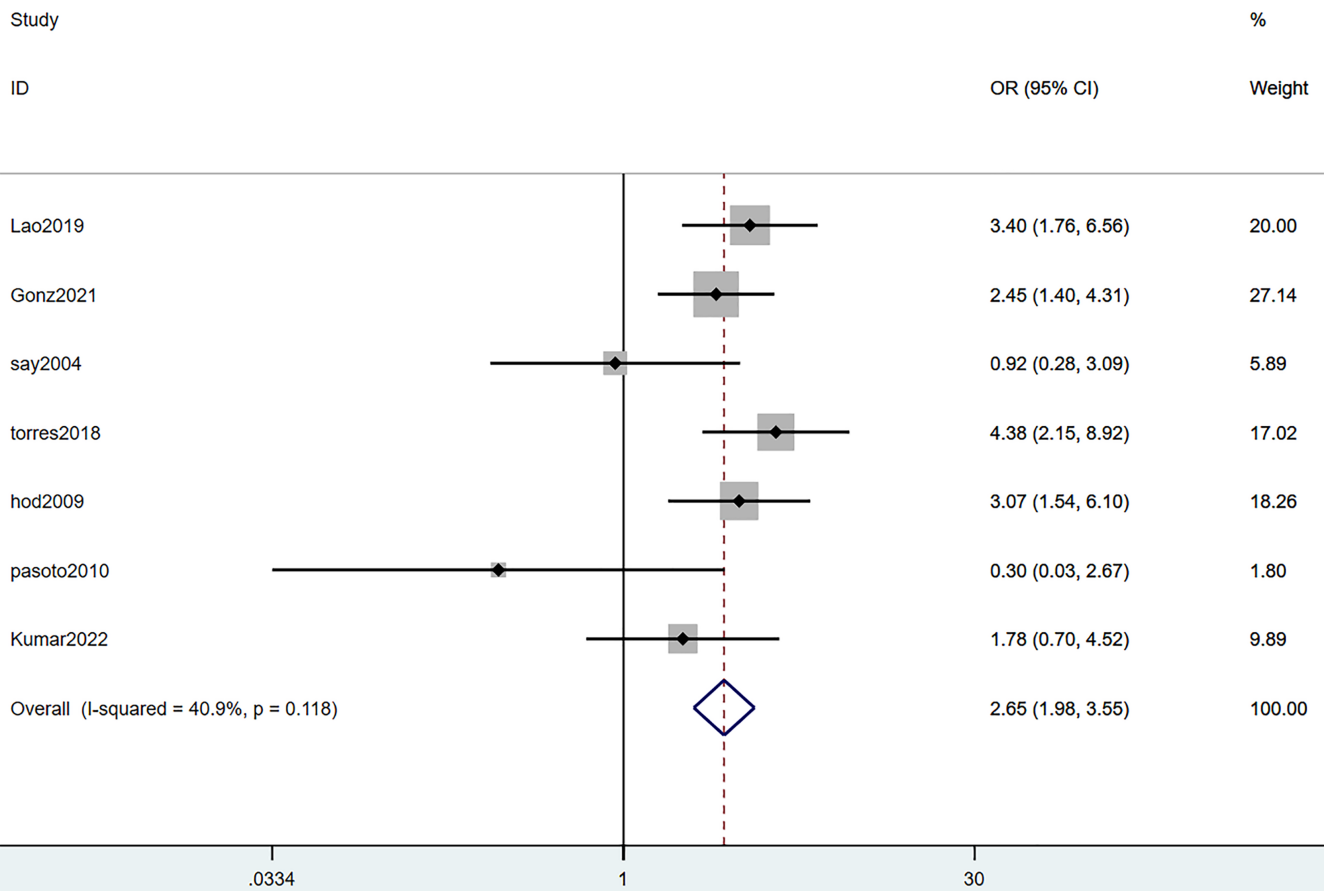
Forest plot of lymphocytopenia (Lao et al., 2019; González-Naranjo et al., 2021; Sayarlioglu et al., 2004; Torres-González et al., 2018; Hodkinson, Musenge & Tikly, 2009; Kumar et al., 2022; Pasoto et al., 2010).

Seven studies reported lymphocytopenia. The heterogeneity test (I^2^ = 40.9%, *P* = 0.118) was performed through a random-effects model. The results revealed that lymphocytopenia was a risk factor for SLE complicated with TB infection, with statistically significant differences (OR = 2.65, 95% CI [1.98–3.55], *P* = 0.000). Details are illustrated in Fig. 3 and Table 3.

#### Anemia

**Figure 4:**
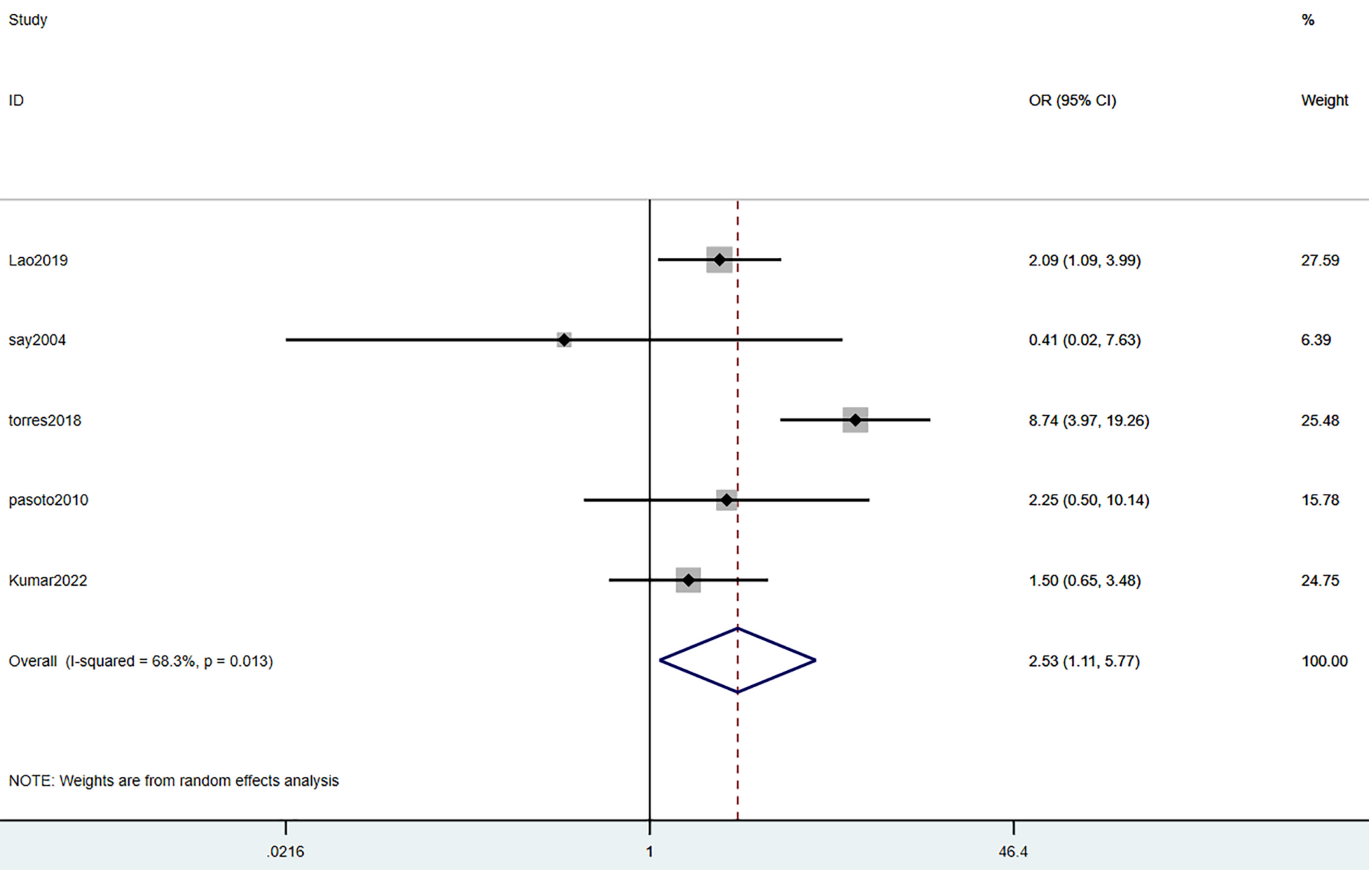
Forest plot of anemia (Lao et al., 2019; Sayarlioglu et al., 2004; Torres-González et al., 2018; Kumar et al., 2022; Pasoto et al., 2010).

Five studies mentioned anemia. The heterogeneity test (I^2^ = 68.36%, *P* = 0.013) was performed through the random-effects model. It was uncovered that anemia was a risk factor for SLE complicated with TB infection, with statistically significant differences (OR = 2.53, 95% CI [1.11–5.77], *P* = 0.001). Details are displayed in Fig. 4 and Table 3.

#### Hypoalbuminemia

**Figure 5:**
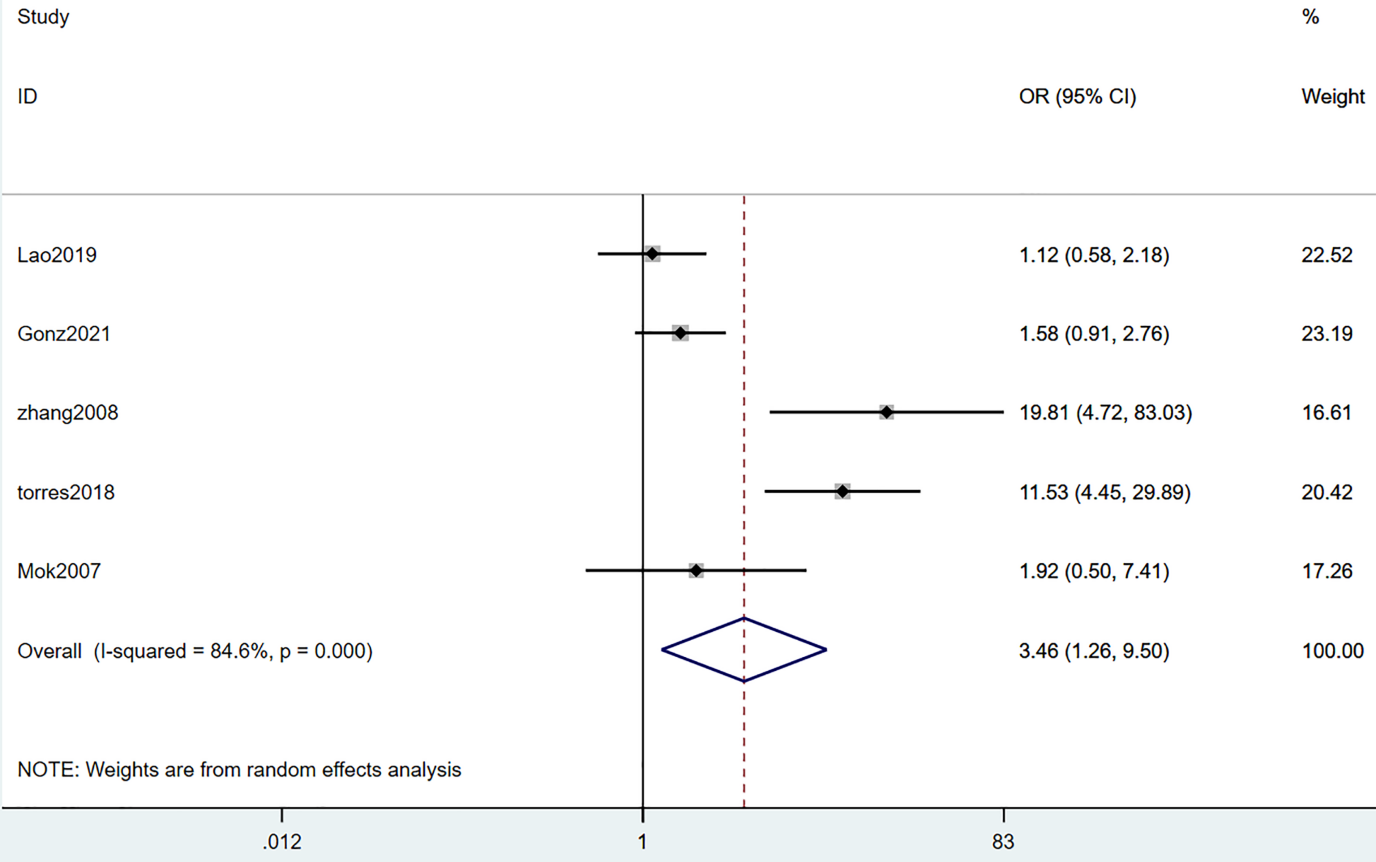
Forest plot of hypoalbuminemia (Lao et al., 2019; González-Naranjo et al., 2021; Torres-González et al., 2018; Mok et al., 2007; Zhang, Wang & Ma, 2008).

Five studies mentioned hypoalbuminemia. The heterogeneity test (I^2^ = 84.6%, *P* = 0.000) was performed through a random-effects model. According to the results, hypoalbuminemia acted as a risk factor for SLE complicated with TB infection, with statistically significant differences (OR = 3.46, 95% CI [1.26–9.50], *P* = 0.016). Details are illustrated in Fig. 5 and Table 3.

#### Diabetes

**Figure 6:**
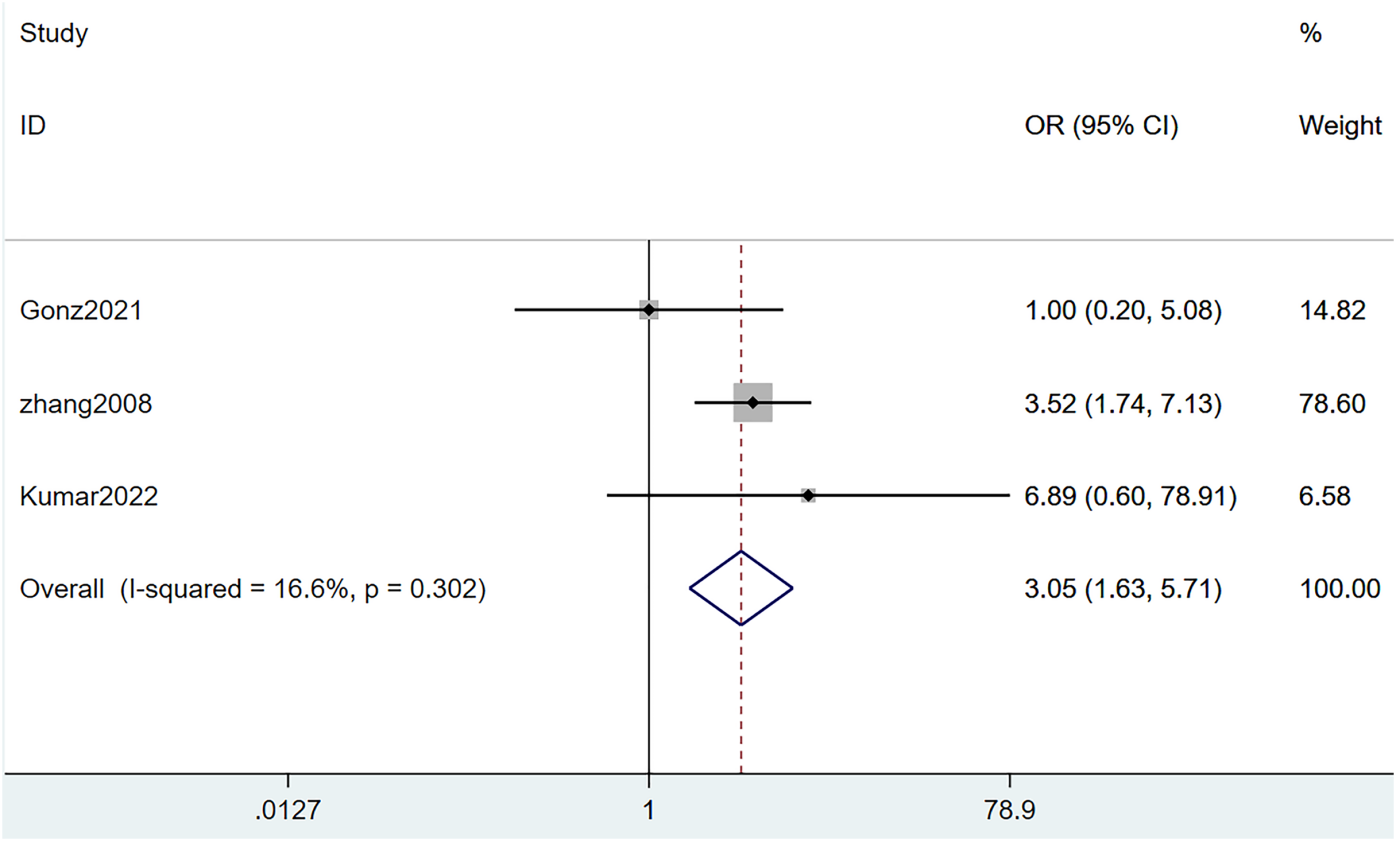
Forest plot of diabetes (González-Naranjo et al., 2021; Kumar et al., 2022; Zhang, Wang & Ma, 2008).

Three studies reported diabetes. The heterogeneity test (I^2^ = 92.4%, *P* = 0.302) was performed through the random-effects model. It was unraveled that diabetes was a risk factor for SLE complicated with TB infection, with statistically significant differences (OR = 3.05, 95% CI [1.63–5.71], *P* = 0.000). Details are illustrated in Fig. 6 and Table 3.

#### Multivariate meta-analysis findings

Meta-analysis of pooled multivariate data demonstrated no significant association between age and SLE-TB comorbidity. The risk factors of SLE complicated with TB infection principally encompassed lymphocytopenia (OR = 2.90, 95% CI [1.89–4.45], *P* = 0.000), cumulative glucocorticoids dosage (OR = 4.88, 95% CI [1.85–12.91], *P* = 0.001), and a history of TB exposure (OR = 3.38, 95% CI [1.16–9.86], *P* = 0.026). Details are illustrated in Table 4.

**Table 4:** Multifactor analysis table.

Risk factors	No of study	Heterogeneity		OR/WMD (95% CI)	*P*	Egger
		I^2^ (%)	*P*			
Multivariate analysis-Lymphocytopenia	4	0.0	0.767	2.90 [1.89–4.45]	0.000	0.313
Multivariate analysis-Accumulative dose of glucocorticoid	4	79.1	0.002	4.88 [1.85–12.91]	0.001	0.202
Multivariate analysis-age	3	88.4	0.000	1.35 [0.93–1.97]	0.118	0.236
Multivariate analysis-History of exposure to tuberculosis	3	86.9	0.000	3.38 [1.16–9.86]	0.026	0.976

#### Other meta-analysis findings

No statistically significant differences were observed and detected in the association between the factors below and SLE complicated with TB infection: age of SLE onset, duration of SLE, age at diagnosis of TB infection, disease activity index, positive antinuclear antibodies to lymphocytopenia, anti-dsDNA, decreased complement C3, lymphocyte count, C-reactive protein, cumulative glucocorticoids, cyclophosphamide, mycophenolate mofetil, methotrexate, cyclosporine A, antimalarials, hematologic disorders, nephritis, discoid rash, photosensitivity, zygomatic rash, neurologic manifestations, musculoskeletal involvement, mucocutaneous involvement, platelet, corticosteroid dose, vasculitis, arthritis, serositis, pleurisy, pulmonary involvement, prednisolone dosage 1 month prior to infection, TB infection-relevant deaths, methylprednisolone pulse therapy within 6 months, history of TB exposure, and other immunosuppressants. Details are specified in Table 3.

### Publication bias

The publication bias was evaluated by using the Egger’s test for each risk factor, applicable to univariate risk factors. Analysis results: male gender (*P* = 0.011), hypoalbuminemia (*P* = 0.016), prednisolone dose at time of infection (10.8–32.5 mg/d) (*P* = 0.007), lymphocytopenia (*P* = 0.000), anemia (*P* = 0.001) showed *P* < 0.05, suggesting publication bias for these indicators. For multivariate analysis results, lymphocytopenia (*P* = 0.000), cumulative glucocorticoid dosage (*P* = 0.001), and history of TB contact (*P* = 0.026) showed *P* < 0.05, suggesting publication bias. Differences were considered statistically significant at *P* < 0.05. For the remaining indicators, *P* > 0.05, suggesting no publication bias, as shown in Tables 3 and 4.

## Discussion

Previous systematic reviews and meta-analyses have elucidated the risk factors for TB infection in SLE (Darmawan et al., 2024), providing important foundation and outlining the disease burden of SLE with comorbid TB. However, this meta-analysis shifts the focus from macroscopic descriptions of epidemiology to the exploration of individualized etiology. More importantly, it reveals the importance of a range of immune and clinical indicators as independent risk factors, particularly establishing the central role of lymphocytopenia. While a previous study (Sundaram et al., 2025) has already explored the relationship between the incidence of SLE and TB infection, providing an overview of the risks associated with different diseases, this meta-analysis specifies and quantifies risk factors within the specific diseases and distinguishes independent influencing factors, providing a critical scientific basis for the precise prevention and control of TB in SLE patients.

A study has established, at a macro level, that SLE patients are at extremely high risk for infections such as TB, a significant public health concern (Pego-Reigosa et al., 2021). Nevertheless, this meta-analysis shifts the focus from group comparisons to analysis of heterogeneity within groups. Using advanced multivariate analysis methods, it reveals key factors determining individual risk, providing direct, robust, and actionable scientific evidence for precise clinical prevention. A epidemiological descriptive study by Wu et al. (2022) lays a solid foundation for understanding the disease burden of TB infection in SLE. Building upon this foundation, our meta-analysis used advanced multivariate analysis to explore individual risk factors, rather than prevalence in populations.

In short, the value of this meta-analysis lies in integrating massive data and extracting relatively independent sets of risk factors through stricter statistical methods (multivariate adjustment), deepening the understanding of TB susceptibility in SLE patients and providing more accurate basis for clinical risk stratification, targeted screening, and prevention. At present, it is believed that the mechanism of SLE combined with TB infection is related to immune abnormalities in SLE patients (Sayarlioglu et al., 2004), which manifest as humoral immune hyperactivity and exacerbate cellular immune deficiencies (Muhammed et al., 2021), making the body susceptible to infection.

As demonstrated in this study, TB contact history is an independent risk factor for SLE patients with TB infection through single and multiple factors, indicating that the risk of TB infection in SLE patients with TB contact history before infection is significantly increased. As a result, active LTBI screening and prevention intervention should be carried out for SLE patients, which will effectively reduce the risk of TB infection and bring significant social benefits (Kocakoc et al., 2003). In traditional research, women were considered a susceptible population for SLE. Nonetheless, this study found that male SLE patients have a significantly increased risk of TB infection, which is more severe (Lee & Song, 2024). This may be related to the mechanism by which androgens indirectly weaken anti TB immunity by regulating Th1/Th2 imbalance (Richard et al., 2001), leading to secondary immunodeficiency and TB infection (Gupta et al., 2022). This finding suggests that clinical screening for TB in male SLE patients needs to be strengthened. Research has found that patients with SLE who use higher doses of cumulative corticosteroids have a significantly increased risk of TB infection. This is due to a decrease in phagocytic cell function, a reduction in NK cell numbers and function, and an increased risk of TB infection mainly caused by cellular immunity. Therefore, during clinical treatment, comprehensive consideration should be given to controlling the dosage of corticosteroids used (Cheng et al., 2019; Muhammed et al., 2021). As a risk factor of SLE complicated with TB infection, diabetes was mentioned in a retrospective analysis study that diabetes may be susceptible to infection with Mycobacterium TB, which may be related to the mechanism that the hyperglycemic environment can promote the proliferation of Mycobacterium TB and inhibit autophagy related clearance (Cheng et al., 2019; Cortes et al., 2008; Hoyt et al., 2019). Therefore, SLE patients using glucocorticoids need to monitor HbA1c and prioritize the use of non insulin sensitizers to control blood sugar, in order to reduce the risk of TB infection (Shah et al., 2022). At the same time, this study also found that hypoalbuminemia, lymphopenia, and anemia are independent risk factors for TB infection in systemic lupus erythematosus. Lower serum albumin levels may impair complement mediated immune responses (Vincent et al., 2003), and are related to the severity and adverse outcomes of the disease (Franco et al., 2024; Yang et al., 2018). Lymphopenia is specifically characterized by increased T cell apoptosis and inhibition of the IL-2 signaling pathway (Cantini et al., 2019; Nesreen, Marwa & Asmaa, 2020), leading to a decrease in TB specific CD4+T cell response ability (Ibrahim, Sani & Timothy, 2016; Scolnik et al., 2014), increasing the susceptibility of SLE patients to TB infection. Anemic SLE patients are prone to TB infection due to a decrease in hemoglobin, which leads to a decline in tissue oxygenation and immune cell function, weakening the ability of macrophages to kill Mycobacterium TB (Dasaradhan et al., 2022). Therefore, during clinical treatment, it is necessary to maintain the patient’s serum albumin and lymphocytes at normal levels as much as possible, and prevent anemia to increase the ability to resist combined TB infection. Early prevention and treatment should be actively carried out to reduce the risk of TB infection after treatment and improve the patient’s prognosis.

This study still has the following limitations: firstly, the number of articles included is relatively small, the analyzed data is insufficient, and there may be selection bias; Secondly, due to differences in research design, population, and diagnostic criteria included in the literature, these may be the reasons and sources of high heterogeneity; Thirdly, due to the retrospective nature of some included cohort studies, there may be selection bias in the results. Therefore, in the future, it is necessary to supplement it with carefully designed prospective studies.

## Conclusion

Based on available evidence, male gender, lymphocytopenia, anemia, hypoalbuminemia, diabetes, prednisolone dosage during infection, current daily prednisone dosage, cumulative glucocorticoids dosage, and a history of TB exposure are risk factors for SLE complicated with TB infection. Clinicians can utilize these indicators for the early detection, diagnosis, and intervention of SLE patients complicated with TB infection. When managing SLE patients, healthcare providers should simultaneously develop rational, standardized TB prevention protocols. Through judicious administration of glucocorticoids and immunosuppressive agents, effective prevention and management of TB infection in SLE patients can be achieved, thereby improving these patients’ quality of life and prognosis.

## Clinical significance

This finding will enable clinicians to better identify the risk of SLE patients with TB infection in practice, from passive response to active attack, accurately identify high-risk groups, avoid missed diagnosis as well as misdiagnosis, force intervention in reversible factors, and prevent infection and achieve early diagnosis and treatment through strict control of hormone accumulation, correction of anemia/low protein, management of diabetes and other ways. Ultimately, through layered prevention and control, the risk of concurrent TB infection in SLE patients is reduced, the condition of patients is improved, medical practice is promoted, and good economic and social benefits are brought about.

## Supplemental Information

10.7717/peerj.20448/supp-1 Supplemental Information 1 PRISMA checklist.

10.7717/peerj.20448/supp-2 Supplemental Information 2 Search strategy.

10.7717/peerj.20448/supp-3 Supplemental Information 3 Systematic Review and/or Meta-Analysis Rationale.

## Additional Information and Declarations

### Data Availability

The following information was supplied regarding data availability:

This is a systematic review/meta-analysis.
