# Supplementary material for: Risk factors for systemic lupus erythematosus complicated with tuberculosis infection: meta-analyses and systematic reviews
Source: PeerJ. 2026 Jan 13;14:e20448. doi: 10.7717/peerj.20448 (PMC12810365; doi:10.7717/peerj.20448)
Supplement: Supplemental Information 2 [file peerj-14-20448-s002.docx]

**Supplementary Material 1** Search strategy

**Index term 1**

system lupus erythematosus

Systemic Lupus Erythematosus

Lupus Erythematosus Disseminatus

Libman-Sacks Disease

Disease, Libman-Sacks

Libman Sacks Disease

**Index term 2**

Tuberculosis infection

Tuberculosis

Tuberculoses

Kochs Disease

Koch's Disease

Koch Disease

Mycobacterium tuberculosis Infection

Infection, Mycobacterium tuberculosis

Infections, Mycobacterium tuberculosis

Mycobacterium tuberculosis Infections

**Index term 3**

risk factors

Factor, Risk

Risk Factor

Social Risk Factors

Factor, Social Risk

Factors, Social Risk

Risk Factor, Social

Risk Factors, Social

Social Risk Factor

Health Correlates

Correlates, Health

Population at Risk

Populations at Risk

Risk Scores

Risk Score

Score, Risk

Risk Factor Scores

Risk Factor Score

Score, Risk Factor
