# Supplementary material for: Risk factors for systemic lupus erythematosus complicated with tuberculosis infection: meta-analyses and systematic reviews
Source: PeerJ. 2026 Jan 13;14:e20448. doi: 10.7717/peerj.20448 (PMC12810365; doi:10.7717/peerj.20448)
Supplement: Supplemental Information 3 [file peerj-14-20448-s003.docx]

## Systematic Review and/or Meta-Analysis Rationale

For systematic reviews / meta-analyses, authors need to provide the following information:

**1.describe the audience it is intended for**

The target audience for this meta-analysis primarily includes the following groups:

1. **Rheumatologists and Researchers**

Focus: The relationship between SLE treatments (e.g., glucocorticoids) and tuberculosis (TB) infection risk, as well as the impact of disease activity and complications on TB susceptibility.

Application: Optimizing treatment plans and identifying high-risk patients for enhanced monitoring.

1. **TB/Infectious Disease Specialists and Researchers**

Focus: Epidemiological characteristics of TB infections in SLE patients, diagnostic challenges (e.g., immunosuppression masking TB symptoms), and preventive strategies.

Application: Developing targeted screening protocols or promoting prophylactic anti-TB therapy.

1. **Public Health Policymakers and Disease Control Agencies**

Focus: The public health burden of TB infections in SLE populations and modifiable risk factors (e.g., living conditions, nutritional status).

Application: Updating TB control guidelines and allocating medical resources (e.g., vaccines, screening tools) in high-risk regions or populations.

1. **Medical Educators and Students**

Focus: Learning meta-analysis methods and the pathological mechanisms linking SLE and TB infection.

Application: Using the study as a teaching case to cultivate evidence-based medicine skills and clinical research design capabilities.

1. **Clinical Guideline Committees**

Focus: Synthesizing evidence to establish recommendations for TB screening and prevention in SLE patients.

Application: Updating international or national guidelines for SLE/TB management (e.g., ACR, WHO guidelines).

This meta-analysis consolidates global evidence to support decision-making across multiple fields, from individualized treatment to public health interventions. The ultimate goal is to reduce TB incidence and mortality in SLE patients. Its conclusions provide guidance for clinical practice, research directions, and policy formulation.

1. **The rationale for conducting the systematic review / meta-analysis.**

**Reply:**

Research background: In recent years, studies have shown that the incidence of tuberculosis infection/tuberculosis in SLE patients is higher than that in the general population. Tuberculosis infection/tuberculosis is one of the most common and deadly infectious diseases in SLE, which can cause severe systemic symptoms in patients, even death and widespread epidemics of the disease.

Insufficiency of existing research: Although previous studies have described the prevalence of tuberculosis infection/tuberculosis in systemic lupus erythematosus through systematic review and meta-analysis, there are currently controversial risk factors for tuberculosis infection/tuberculosis in systemic lupus erythematosus patients. There is no consensus on the risk factors and there is a lack of evidence-based medical support.

Necessity of this study: Considering the early onset and high prevalence of SLE, determining the risk factors for tuberculosis infection/tuberculosis is one of the important issues that must be treated promptly and appropriately, which has a serious impact on the outcome of the disease. Although several studies have attempted to identify these risk factors, many results have been found to be inconsistent due to the heterogeneity and complexity of SLE. From this perspective, this study provides a systematic review and meta-analysis to summarize and elucidate the risk factors for TB infection/TB disease in SLE patients.

1. **The contribution that it makes to knowledge in light of previously published related reports, including other meta-analysesnd systematic reviews.**

**Reply:**

Clinical significance: Meta-analysis of risk factors can integrate data from multiple studies and identify the core risk factors for tuberculosis infection in SLE patients. Clinicians can use this to stratify patients, strengthen monitoring (such as regular tuberculosis screening) or take preventive measures for high-risk groups, optimizing clinical treatment plans and providing a basis for drug selection. Identifying risk factors can help identify tuberculosis infection early, thereby reducing mortality. This study can guide screening strategies to combine regional epidemiological data (such as high TB burden areas) and determine the applicable population for preventive treatment.

Scientific research significance: This study solves research heterogeneity. Single studies often lead to inconsistent conclusions due to small sample sizes, population differences or regional biases. Meta-analysis can improve statistical power and identify truly universal risk factors by quantitatively integrating data. It provides direction for subsequent basic research, support guideline formulation and policy adjustment results to provide evidence for the update of international or regional clinical guidelines, especially in areas with high tuberculosis incidence.

Summary: Meta-analysis system to evaluate the risk factors of SLE combined with tuberculosis infection can not only optimize clinical decision-making and improve patient prognosis, but also promote the study of pathogenesis, fill scientific research gaps, and promote multidisciplinary collaboration (such as joint management of rheumatology and infectious diseases). In the future, attention should be paid to the inclusion of high-quality prospective studies, regional diversity analysis, and in-depth exploration of molecular risk factors. When treating SLE patients, clinicians should formulate reasonable and standardized tuberculosis prevention strategies, and achieve the prevention and treatment of SLE combined with tuberculosis infection/tuberculosis through the rational use of glucocorticoids, thereby improving the quality of life of such patients and improving their prognosis.
